# Isolation, biochemical characterization, and greenhouse authentication of chickpea *(Cicer arietinum L.)* rhizobia collected from some major chickpea growing areas of Woldia, North Wollo, Ethiopia

**DOI:** 10.1371/journal.pone.0330169

**Published:** 2025-08-14

**Authors:** Kelemu Yimenu Gebre, Abebe Girma Demissie, Aderajew Adgo Tesema, Habtamu Zegeye Belay, Mekashaw Worku Akalye, Agere Bereket Friew, Fisseha Getachew Baye, Habtie Bassie Felatie, Mezgebu Abunie Yohannes, Marye Alemu Eshetu, Wondye Ayalew Shiferaw

**Affiliations:** Department of Biotechnology, College of Natural and Computational Science, Woldia University, Ethiopia; Universiti Putra Malaysia (UPM), MALAYSIA

## Abstract

Chickpea (*Cicer arietinum L.*) is a vital legume crop worldwide, valued for its high nutritional content and significant contribution to food security and soil fertility through biological nitrogen fixation. Despite its importance, chickpea yields remain suboptimal in many regions, including Ethiopia, primarily due to constraints such as poor soil fertility and inadequate use of effective rhizobia inoculants. This study aimed to isolate and characterize native Rhizobium strains from chickpea root nodules collected from fields in the Woldia region and to assess their potential to promote plant growth. A total of 41 bacterial isolates were obtained, of which 12 were presumptively identified as Rhizobium based on growth characteristics on Congo red and bromothymol blue media. These isolates were further characterized morphologically and biochemically. Five biochemically promising isolates were selected for evaluation in a controlled 45-day greenhouse experiment under sterile conditions. Inoculation with these isolates significantly enhanced seed germination and early seedling growth compared to uninoculated controls. The symbiotic effectiveness of the isolates ranged from 74.3% to 121.9%, with isolates WUSFDG-23, WUSFMC-31, and WUSFMC-23 demonstrating high effectiveness, isolate WUSFDG-23 markedly increased nodulation and biomass accumulation. This study highlights the potential of native Rhizobium isolates from Woldia chickpea fields, especially WUSFDG-23, as effective bio-inoculants to promote sustainable chickpea production and reduce dependence on chemical fertilizers.

## Introduction

Chickpea (*Cicer arietinum L*.) is a major pulse crop cultivated globally, including in Ethiopia, where it plays a vital role in household nutrition, food security, and income generation. It ranks as the third most important legume export in Ethiopia after faba bean and haricot bean, contributing approximately USD 61 million annually [1,2]. Despite its economic and nutritional significance, chickpea productivity remains suboptimal in many Ethiopian regions, such as Woldia, primarily due to low soil fertility and inadequate use of sustainable nutrient management practices [3,4].

The widespread reliance on chemical nitrogen fertilizers to enhance crop yields has led to several agronomic and environmental challenges. Excessive fertilizer use contributes to soil acidification, nutrient imbalances, environmental pollution, and increased production costs, which disproportionately affect smallholder farmers [5,6]. These issues underscore the urgent need for environmentally sustainable and cost-effective alternatives to chemical fertilizers, such as plant growth-promoting bacteria (PGPB) and symbiotic nitrogen-fixing rhizobia [7,8].

Rhizobia are Gram-negative soil bacteria capable of establishing a symbiotic relationship with leguminous plants, including chickpea, by fixing atmospheric nitrogen (N₂) into ammonia (NH₃), a form directly usable by plants [9,10]. This symbiosis is initiated when legume roots exude flavonoids and other chemical signals, which are recognized by rhizobia, triggering the production of nodules. These signaling molecules induce root hair curling, infection thread formation, and development of root nodules, specialized organs where nitrogen fixation occurs under microaerobic conditions [11,12].

Inside these nodules, rhizobia differentiate into bacteroids and express nitrogenase enzymes that catalyze the conversion of atmospheric nitrogen into ammonia. Although this process is energetically costly, it is supported by carbohydrates supplied by the host plant through photosynthesis, forming a mutually beneficial interaction [13,14]. Fixed nitrogen is essential for synthesizing amino acids, proteins, nucleotides, and vitamins, thereby supporting plant growth and productivity [15]. Besides nitrogen fixation, rhizobial symbiosis improves soil structure and fertility, promoting sustainable agriculture and reducing the dependence on synthetic fertilizers [12,9]. In Ethiopia, despite the potential benefits, the use of rhizobial inoculants is limited by the lack of commercial inoculant production facilities, poor farmer awareness, and the absence of effective native strains adapted to local agro-ecological conditions [4,6]. Native rhizobia often demonstrate superior symbiotic efficiency and environmental resilience compared to exotic strains, making them ideal candidates for developing biofertilizers tailored to Ethiopian soils and climates [7,10]. This study aims to isolate and characterize native rhizobial isolates from chickpea root nodules collected around Woldia. It also seeks to evaluate their symbiotic efficiency and potential as bio-inoculants to enhance chickpea growth and biological nitrogen fixation in a greenhouse.

## Materials and methods

### Description of study area

This study was conducted in the biotechnology laboratory at Woldia University. Woldia is approximately 521 km north of Addis Ababa and lies at an elevation of about 2,380 meters above sea level. The town is positioned at approximately 11°50′N latitude and 39°36′E longitude. Woldia is characterized by mountainous terrain and falls within the subtropical highland climate zone. The area experiences moderate temperatures ranging from 10°C to 25°C annually, with an average annual rainfall of 750–1,200 mm, predominantly occurring from June to September [16]. The climate supports a diverse range of agricultural practices and significantly contributes to the livelihoods of the local population. The town has a population of over 90,000 residents and serves as a key administrative and commercial center in the North Wollo Zone. The local economy is predominantly agrarian, with mixed crop-livestock farming being the primary agricultural system. Major crops cultivated in the area include chickpea, wheat, barley, teff, sorghum, and maize, while common legumes include lentils and faba beans. Livestock such as cattle, sheep, goats, donkeys, and poultry are also widely reared.

The soils in and around Woldia are primarily Vertisols and Cambisols, which are moderately fertile and suitable for legume cultivation, particularly chickpeas. These soil types generally exhibit good moisture retention properties but may vary in texture and organic matter content depending on specific topographical and land use conditions.

The specific sampling sites for root nodule collection, along with their geographic coordinates and altitudes, are provided in Table 1 below:

**Table 1:** Geographic coordinates and altitudes of root nodule sampling sites.

No.	Sampling site	Zone	District	Latitude (N)	Longitude (E)	Altitude
1	WUSFME-1	N/Wollo	Mersa	11° 39’ 53.84“N	39° 39’ 34.41“E	1600 m
2	WUSFME-2	N/Wollo	Mersa	11° 40’ 10.25“N	39° 39’ 20.18“E	1610 m
3	WUSFME-3	N/Wollo	Mersa	11° 39’ 35.70“N	39° 39’ 45.50“E	1595 m
4	WUSFMC-1	N/Wollo	Mechare	11.8028° N	39.5729° E	1774 m
5	WUSFMC-2	N/Wollo	Mechare	11.8045° N	39.5742° E	1768 m
6	WUSFMC-3	N/Wollo	Mechare	11.8000° N	39.5705° E	1782 m
7	WUSFSI-1	N/Wollo	Sirinka	11.7560° N	39.6045° E	2106 m
8	WUSFSI-2	N/Wollo	Sirinka	11.7582° N	39.6060° E	2110 m
9	WUSFSI-3	N/Wollo	Sirinka	11.7548° N	39.6031° E	2098 m
10	WUSFDG-1	N/Wollo	Doro Gibir	11° 52’ 0“N	39° 40’ 0“E	1532 m
11	WUSFDG-2	N/Wollo	Doro Gibir	11° 51’ 45“N	39° 40’ 15“E	1526 m
12	WUSFDG-3	N/Wollo	Doro Gibir	11° 52’ 20“N	39° 39’ 50“E	1540 m

Key; WU-Woldia University, SFDG-Sample from Doro Gibir, SFMC- Sample from Mechare, SFME-Sample from Mersa, SFSI-Sample from Sirina.

### Sample collection

Twelve root nodule samples were collected from chickpea-growing areas around Woldia, such as Sirinka, Mersa, Mechare, and Doro Gibir, using a random sampling method. Sampling points were located at least 5 meters away from the field edges to minimize potential edge effects. Soil and root nodules were collected at an approximate depth of 20 cm using sterile soil and hand tools, such as spatulas, while wearing gloves to prevent contamination. Samples were placed into sterile polythene bags, labelled, and transported in a clean icebox to the laboratory. Upon arrival, samples were stored at 4ºC until processing. Fresh, healthy root nodules were selected based on their light brown or pinkish colour, indicating active nitrogen fixation between the rhizobia and chickpea plants [17].

A total of 24 pots were arranged in a Completely Randomized Design (CRD) with eight treatments and three replications each. The treatments included a negative control (dH₂O, no nitrogen or inoculum), a positive control (20 kg N ha ⁻ ¹ as urea, without inoculation), a commercial inoculant (SRCP17 from the Soil Science Research Centre, Dessie), and five isolates WUSFSI-21 (isolated from Sirinka), WUSFDG-23 (isolated from Doro Gibir), WUSFME-21 (isolated from Mersa), WUSFMC-31, and WUSFMC-23 (isolated from Mechare). Each pot was filled with sterilized sand soil and seeded with surface-sterilized chickpea seeds, with seeds in the inoculated treatments with their respective isolates. Pots were irrigated every two days with 200 mL of distilled water to maintain approximately 60% of field capacity, ensuring consistent moisture without waterlogging. After 45 days of growth, data were collected on shoot length, root length, nodule number, nodule dry weight, shoot dry weight, and symbiotic effectiveness, enabling a clear comparison of the growth-promoting potential and nitrogen fixation efficiency among the different treatments.

### Media preparation

The nutrient medium used for isolation of the rhizobia was yeast extract mannitol (YEM) agar, with the following composition: mannitol 10 gm/l, anhydrous magnesium sulfate 0.2 gm/l, sodium chloride 0.1 0.1 gm/l, di-potassium hydrogen phosphsate 0.5 0.5 gm/l, anhydrous calcium chloride 0.2 0.2 gm/l, anhydrous iron chloride 0.01 0.01 gm/l, yeast extract 1.00 1.00 gm/l, agar 20 gm/l, and distilled water1000 ml [18]. And the composition of Yeast Mannitol Broth (YMB) was 10 mg/l mannitol, 200 mg/l anhydrous magnesium sulfate, 100 mg/l sodium chloride, 500 mg/l di-potassium hydrogen phosphate mg/l yeast extract, and 1000 ml distilled water [19]. Each of the components was measured with a weighing balance and put into a conical flask containing distilled water, and then covered with aluminum foil. The pH of the medium was adjusted to 7 using a pH meter after this process. The medium was autoclaved at a temperature of 121ºC for 15 min and allowed to cool, and was poured into Petri dishes (approx. 20 ml in each) aseptically in the hood. This solidified medium can be used for the enumeration of symbiotic rhizobia microbes by using spread plate techniques.

### Isolation of rhizobia isolates

Initially, chickpea root nodules were detached and washed under running tap water to remove adhering soil particles. Intact and undamaged nodules were then carefully excised from the roots using sterile forceps. To ensure surface sterilization, the nodules were immersed in 95% ethanol for 5–10 seconds to break the surface tension, followed by soaking in a 3% hydrogen peroxide (H₂O₂) solution for 2–3 minutes. The sterilized nodules were then rinsed thoroughly in five successive changes of sterile distilled water.

Sterilized nodules were aseptically crushed using a sterile mortar and pestle to obtain a milky suspension of bacteroids [17]. The suspension was streaked onto yeast extract mannitol agar (YEMA) medium containing ketoconazole (20.0 μg/ml) to reduce fungal contamination. Petri plates were incubated at 28 °C for 48 hours. After incubation, well-isolated, typical rhizobial colonies characterized by their white, circular, convex, and mucoid appearance were selected and subcultured onto fresh YEMA plates using the streak plate method to obtain pure cultures. All isolation and subculturing steps were carried out in a laminar airflow cabinet to maintain aseptic conditions [20]. Only morphologically distinct and purified colonies consistent with rhizobia characteristics were selected for further biochemical and physiological characterization.

### Preliminary tests of rhizobia

Two different preliminary tests (growth on YEMA with Congo red and bromothymol blue) were performed to confirm the isolate as rhizobia and to differentiate them from other microbes [21].

### Bromothymol blue test

Rhizobia were cultivated on YEM agar medium supplemented with 0.025% bromothymol blue for this test, and the culture was kept at 28°C for 48 hours. In this test sample, YEM medium with BTB was allowed to grow. The formation of acid caused the positive sample to turn yellow after 48 hours of incubation at 28°C [20].

### Congo red absorption test

This was done to confirm the purity of rhizobia isolates. Congo red stock solution was prepared by dissolving 2.5 g Congo red in 1000 mL sterilized distilled water. Ten (10) milliliters of Congo red was added to a liter of YEMA media and sterilized. Finally, a loop full of test isolates was streaked on the medium, covered with aluminum foil, and incubated at 28 ± 2°C for 72 hours. The absorption of Congo red by the rhizobia isolates, which indicated contaminant growth, was recorded. Isolates that did not absorb the Congo red were selected for future purification work [22].

### Purification and preservation of isolates

Using a sterile inoculating loop, isolated colonies were selected, streaked on sterile YEMA plates, and then incubated at 28 ± 2^o^C. Through serial re-streaking and the selection of a single well-isolated colony to a YEMA slant containing 0.3% (w/v) CaCO₃ in a culture tube and incubation at 28 °C, the purity and homogeneity of the colony type were meticulously investigated. The culture slant was kept at 4°C after it had grown sufficiently [23]. The isolates were streaked on YMA slants for long-term storage, and sterile glycerol was added to the media [17].

### Morphological characterization

Each isolate was inoculated on YEMA with a loopful of a 48-hour broth culture (Yeast Extract Mannitol Broth (YEMB)) and incubated for 48 hours at 28 °C. Cell shape, Gram staining, and other colony traits were used to define individual colonies. Type of growth, colony color, elevation, borders, texture, and colony size were the morphological observations [24].

### Gram staining of the rhizobia isolates

The tests were conducted by using a loopful of pure culture grown on YEMA was then put in for Gram staining for more specific identification of the colonies. The Gram staining was done in a laminar air flow hood. Using a clean slide and sterile wire loop, a loopful of normal saline was dropped at the center of the slide, and the loop was sterilized again. The specimen was collected with the wire loop. A smear was made circularly. After making the smear, it was heat-fixed on the slide by passing it gently over the Bunsen flame. Crystal violet was put on the smear for 30 seconds and poured off. Iodine solution was poured on and left for 1 min. It was then washed off with tap water. Then, ethanol was added for decolonization purposes, and subsequently, the ethanol was poured off, and water was poured immediately. Counterstaining using Safranin was done with water within a minute. After drying the slide surface, observe it through a compound microscope. Finally, immersion oil was added and viewed with a 100x oil immersion objective lens [25].

### Biochemical characterization of isolates

#### Motility test.

Using a sterilized straight wire, rhizobia isolates were injected into a test tube with a SIM medium containing 0.5% YEMA. The test tube was then incubated at 28°C, pH 7.0, and incubated for 48 hours. If the isolates moved away from the line of inoculation, it was a positive result, suggesting motility; if not, it was a negative result, indicating non-motility [24].

#### Starch hydrolysis test.

These experiments were conducted to ascertain rhizobia’s capacity to use starch as a source of carbon. The test microorganisms in the starch hydrolysis test were cultivated on agar plates that contained 1% starch. The bacteria were cultivated on the hood’s supplied media and incubated at 32 °C, pH 7.0, and for 48 hours. Following the appearance of bacterial colonies, a drop of iodine (0.1N) was applied to a culture that had been incubated for 48 hours and had a distinct zone of inhibition surrounding the colonies. Following the incubation period, a drop of iodine solution was added; the development of a clear zone signifies a successful starch hydrolysis test [26].

#### Catalase test.

The purpose of this test was to determine whether the examined organism contained the catalase enzyme. One loopful of the rhizobia isolates was suspended in one milliliter of 3% hydrogen peroxide on a glass slide to test for catalase activity. According to the usual procedure, these tests were conducted [17]. The formation of bubbles indicates a positive catalase test result.

#### Oxidase test.

The presence of the oxidase enzyme in several isolates was assessed using the oxidase test. Kovac’s reagent (1% N-tetramethylphenylene diamine) was dissolved in warm water and stored in a dark bottle. One-day-old rhizobia colonies from agar plates were placed on a strip of filter paper that had been dipped in this reagent and allowed to air dry. Within five minutes, oxidase-positive colonies changed from lavender to dark purple to black [20].

#### Triple sugar iron (TSI) agar test.

Triple sugar iron agar medium was used to inoculate test tubes with cultures of isolates that were actively growing. The test was conducted using the stab-and-streak method by incubating at 28°C, pH 7.0, for 48 hours. Through a shift in the butt and slant colours, the test was conducted to determine the isolates’ capacity to ferment different sugars, such as lactose, glucose, and sucrose. Following incubation, colour was seen on the slant and butt. Three observations might be made based on the bacterial isolate’s capacity to consume carbohydrates. An alkaline/alkaline (red slant/red butt) shows the absence of carbohydrate fermentation, an acid/acid (yellow slant/yellow butt) indicates the fermentation of lactose and sucrose, and an alkaline/acid (red slant/yellow butt) indicates the fermentation of dextrose exclusively [27].

#### Citrate utilization test.

The purpose of this test was to confirm whether or not the test organism used citrate as a carbon source. The test was conducted on Simmons’ citrate agar slants by streaking the isolates onto Simmons’ citrate agar slants and incubated at 28°C, pH 7.0, for 48 hours. According to Hamza *et al.* [25], a noticeable shift in colour from green to blue indicates a positive citrate utilization test result.

#### Gelatin hydrolysis.

The purpose of the test was to ascertain whether the isolates could use gelatin as a media source and create the proteolytic enzyme gelatinase. The presence of the enzyme gelatinase is shown by the degradation of gelatin. The nutritional gelatin stab method is the most widely used and standard technique. After being stab-inoculated at 28 °C for 48 hours at pH of 7.0, in nutritional gelatin medium (5 g/L peptone, 3 g/L beef extract, 12 g/L gelatin, 1000 ml of distilled water). The culture was put at a low temperature of 4°C for 30 minutes [19].

#### Urease test.

The rhizobia isolates were inoculated individually into test tubes with urea agar Slant (K_2_HPO_4_ 2 g. L^-1^, urea 20 g. L^-1^, phenol red 0.012 g. L^-1^, agar 15 g. L^-1^, pancreatic digest of gelatine 1 g. L^-1^, dextrose 1 g. L^-1^, and 1 L dis. H_2_0) and incubated at 28°C for 48–48 hours. Following the incubation period, the isolates’ positive urease activity is shown by the emergence of a deep pink colour [27].

#### Nitrogen fixation test.

To qualitatively assess the nitrogen-fixation potential of the isolates, a nitrogen-free (N-free) medium was prepared based on the formulation described by Rodrigues *et al*. [28], with slight modifications. The medium contained the following components per liter of distilled water: 0.2 g magnesium sulfate heptahydrate (MgSO₄·7H₂O), 0.2 g sodium chloride (NaCl), 0.1 g ferrous sulfate heptahydrate (FeSO₄·7H₂O), 5 g sodium molybdate dihydrate (Na₂MoO₄·2H₂O), 1 g calcium carbonate (CaCO₃), 1 g dipotassium hydrogen phosphate (K₂HPO₄), and 15 g agar. The pH of the medium was adjusted to 7.0 using 1 N NaOH a HCl before sterilization by autoclaving at 121 °C for 15 minutes and incubated at 28 °C for five days.

#### Indole test.

Tryptophan broth medium was prepared and poured into 10 mL test tubes and then autoclaved at 121°C for 15 min. rhizobia were inoculated at 28°C with a pH of 7.0 for 48 hours. An un-inoculated broth served as the control. After incubation, 1 mL of Kovac’s reagent (isoamyl alcohol, *para*-dimethyl amino benzaldehyde, and concentrated hydrochloric acid) was added to each test tube, including the control. Tubes were shaken gently and allowed to stand until the reagent surfaced. The formation of a red ring indicated a positive result, but a yellow ring indicated a negative result [29].

#### Ammonia (NH_3_) production.

A loopful of newly developed cells was inoculated into 10 ml of sterile peptone water (pH 7.0) and the isolates were then incubated at 28°C for 72 hours to test for the production of ammonia. To identify the formation of a brown to yellow colour as a positive test for ammonia generation, the tubes were treated with 0.5 ml of Nessler’s reagent (potassium iodide 50 g, saturated mercuric chloride 35 mL, distilled water 25 mL, potassium hydroxide (40%) 400 mL) [30].

#### Effect of rhizobia on seed germination.

Seed germination studies were conducted using the water agar method. Chickpeas were surface sterilized with 70% ethanol and then treated with 1% calcium hypochlorite for 2 min, followed by repeated washing with sterile water. After this, the seeds were soaked in the rhizobial culture broth, while seeds that were soaked in normal nutrient broth were kept as a control. A final concentration of 10^8^ CFU mL ⁻ ¹, then coated onto the surface-sterilized chickpea seeds and allowed to air dry. Ten seeds of each treatment were kept equidistant in sterilized Petri plates containing moist filter paper with three replicates for each treatment, and the Petri plates were incubated at room temperature (25°C). Germination counting began after 24 hours of sowing and continued until the 15^th^ day. Seed germination and percent seedling emergence were calculated using the following formula: % seed emergence = number of emerged seedlings/number of seeds sown × 100 [31]. After 2 weeks, the germination, secondary root number and primary root length were measured and recorded [32]. Seedling vigour index = % germination × (seedling dry weight) [33].

#### Symbiotic effectiveness screening at the greenhouse.

The experiment was conducted in a greenhouse at Wolda University Botanical Garden. A total of 24 pots were set up in a completely randomized design (CRD), each containing eight different treatments: five rhizobial inoculants, namely WUSFSI (from Sirinka), WUSFDG (from Doro Gibir), WUSFME (from Mersa), WUSFMC (from Mechare), CP17 (a commercial inoculum from Desse soil science); N fertilizer (20 kg N ha^-1^, without inoculant); and distilled water (neither fertilizer nor inoculation). The study was organized with three replications in a completely random design within the greenhouse. 3 kg of sterilized sand soil was placed into surface-sterilized plastic pots [33] with a capacity of five kg. The chickpea (*Cicer arietinum* L.) cultivar “Metek,” sourced from the Sirinka Agricultural Institute, was surface sterilized using 95% ethanol for 10 seconds and 3% sodium hypochlorite for 3 minutes, followed by rinsing with sterile water. The seeds were allowed to dry in a laminar airflow for a few minutes. Five surface-sterilized seeds were germinated on sterilized water agar plates (7.5 g of agar in 1,000 ml of distilled water) at room temperature(25°C) for 48 hours. Five uniform, undamaged seeds were selected and transferred into each pot containing sterilized sand using sterile forceps. Three days post-germination, each seedling received 1 ml of a 72-hour YEMB culture (approximately 1 ml of each *Rhizobium* isolate at 1 × 10^8^cfu/ml) applied around the root with a sterile pipette and covered with a layer of sterile sand soil. One week after germination, the seedlings were thinned to maintain three healthy plants per pot. All plants were given 100 ml of quarter-strength N-free nutrient solution(with the composition of:Calcium chloride dihydrate (CaCl₂·2H₂O) 0.025 g/L, Magnesium sulphate heptahydrate (MgSO₄·7H₂O) 0.025 g/L, Monopotassium phosphate (KH₂PO₄) 0.025 g/L, Dipotassium phosphate (K₂HPO₄) 0.025 g/L, Boric acid (H₃BO₃) 0.000715 g/L, Manganese sulphate monohydrate (MnSO₄·H₂O) 0.0005575 g/L, Zinc sulphate heptahydrate (ZnSO₄·7H₂O) 0.000055 g/L, Copper sulphate pentahydrate (CuSO₄·5H₂O) 0.00002 g/L, Sodium molybdate dihydrate (Na₂MoO₄·2H₂O) 0.0000975 g/L, Iron (Fe) as Fe-EDTA 0.0025 g/L.) weekly, as described by Somasegaran and Hoben [34]. The pots were watered every two days [35].

#### Symbiotic effectiveness of isolates.

Plants were uprooted forty-five days post-planting to measure shoot length, nodule number, nodule dry weight, and shoot dry weight. Before measurement, roots and nodules were carefully cleaned using running tap water to remove adhering soil particles, ensuring accurate assessment of biomass. The symbiotic effectiveness was calculated using the following formula: % SE = Inoculated plants SDW/N fertilized plants SDW × 100, where SDW = shoot dry weight, N = nitrogen, and SE = symbiotic effectiveness. High efficacy (≥80%), low effectiveness (35–49%), effective (50–79%), and ineffective (<35%) were the rates of nitrogen-fixing effectiveness [21,36].

#### Data analysis.

All experimental data were collected in triplicate, and the results were presented. Statistical analyses were conducted with Statistic 10 and SPSS (version 27.0.1). The mean values comparison was performed following the significance test results from One-way ANOVA using least significant difference (LSD) at a 5% level of probability. The LSD test also provides clearer interpretability when assigning statistical groupings using letter notations. Differences between means were considered statistically significant at a 5% probability level (p ≤ 0.05). Significant variations between treatments were indicated by different lowercase letters assigned to each mean in the data tables. The correlation between different parameters was evaluated by using the Pearson correlation coefficient.

## Results

### Isolation, screening, and morphological characterization of rhizobia

In this study, the isolates were obtained from root nodules of chickpea plants collected from different chickpea-growing areas. A total of forty-one isolates were successfully cultured on Yeast Extract Mannitol Agar (YEMA) medium. All isolates were subjected to preliminary morphological examination based on colony and cell characteristics. Across all samples, the colonies demonstrated remarkably consistent morphological traits. The colonies appeared white, with a circular shape and entire margins. They exhibited a mucoid texture, a typical feature of rhizobial exopolysaccharide production, and seemed transparent to translucent in optical appearance. The colony surface was uniformly smooth, indicating healthy, non-filamentous bacterial growth. Microscopic examination using Gram staining revealed that all 41 isolates were Gram-negative (-ve) and rod-shaped (Table 2), further supporting their classification within the *Rhizobium* genus. This uniformity in colony and cell morphology across all isolates suggests that the isolates are likely to be members of the same or closely related species of rhizobia. However, further physiological and biochemical tests were conducted to support this preliminary identification and to assess potential variability in functional traits.

**Table 2:** Morphological characteristics of 41 rhizobia isolates.

Isolate Code	CC	CF	CT	O	CM	A	GS	SB
WUSFSI-11	White	Circular	Mucoid	Transparent	Entire	Smooth	-ve	Rod
WUSFSI-11	White	Circular	Mucoid	Transparent	Entire	Smooth	-ve	Rod
WUSFSI-12	White	Circular	Mucoid	Transparent	Entire	Smooth	-ve	Rod
WUSFSI-13	White	Circular	Mucoid	Transparent	Entire	Smooth	-ve	Rod
WUSFSI-13	White	Circular	Mucoid	Transparent	Entire	Smooth	-ve	Rod
WUSFSI-21	White	Circular	Mucoid	Transparent	Entire	Smooth	-ve	Rod
WUSFSI-22	White	Circular	Mucoid	Transparent	Entire	Smooth	-ve	Rod
WUSFSI-23	White	Circular	Mucoid	Transparent	Entire	Smooth	-ve	Rod
WUSFSI-31	White	Circular	Mucoid	Transparent	Entire	Smooth	-ve	Rod
WUSFSI-33	White	Circular	Mucoid	Transparent	Entire	Smooth	-ve	Rod
WUSFME-11	White	Circular	Mucoid	Transparent	Entire	Smooth	-ve	Rod
WUSFME-11	White	Circular	Mucoid	Transparent	Entire	Smooth	-ve	Rod
WUSFME-12	White	Circular	Mucoid	Transparent	Entire	Smooth	-ve	Rod
WUSFME-13	White	Circular	Mucoid	Transparent	Entire	Smooth	-ve	Rod
WUSFME-13	White	Circular	Mucoid	Transparent	Entire	Smooth	-ve	Rod
WUSFME-21	White	Circular	Mucoid	Transparent	Entire	Smooth	-ve	Rod
WUSFME-21	White	Circular	Mucoid	Transparent	Entire	Smooth	-ve	Rod
WUSFME-22	White	Circular	Mucoid	Transparent	Entire	Smooth	-ve	Rod
WUSFME-23	White	Circular	Mucoid	Transparent	Entire	Smooth	-ve	Rod
WUSFME-23	White	Circular	Mucoid	Transparent	Entire	Smooth	-ve	Rod
WUSFME-31	White	Circular	Mucoid	Transparent	Entire	Smooth	-ve	Rod
WUSFMC-11	White	Circular	Mucoid	Transparent	Entire	Smooth	-ve	Rod
WUSFMC-12	White	Circular	Mucoid	Transparent	Entire	Smooth	-ve	Rod
WUSFMC-21	White	Circular	Mucoid	Transparent	Entire	Smooth	-ve	Rod
WUSFMC-23	White	Circular	Mucoid	Transparent	Entire	Smooth	-ve	Rod
WUSFMC-31	White	Circular	Mucoid	Transparent	Entire	Smooth	-ve	Rod
WUSFMC-32	White	Circular	Mucoid	Transparent	Entire	Smooth	-ve	Rod
WUSFMC-33	White	Circular	Mucoid	Transparent	Entire	Smooth	-ve	Rod
WUSFDG-11	White	Circular	Mucoid	Transparent	Entire	Smooth	-ve	Rod
WUSFDG-12	White	Circular	Mucoid	Transparent	Entire	Smooth	-ve	Rod
WUSFDG-12	White	Circular	Mucoid	Transparent	Entire	Smooth	-ve	Rod
WUSFDG-13	White	Circular	Mucoid	Transparent	Entire	Smooth	-ve	Rod
WUSFDG-13	White	Circular	Mucoid	Transparent	Entire	Smooth	-ve	Rod
WUSFDG-21	White	Circular	Mucoid	Transparent	Entire	Smooth	-ve	Rod
WUSFDG-22	White	Circular	Mucoid	Transparent	Entire	Smooth	-ve	Rod
WUSFDG-23	White	Circular	Mucoid	Transparent	Entire	Smooth	-ve	Rod
WUSFDG-23	White	Circular	Mucoid	Transparent	Entire	Smooth	-ve	Rod
WUSFDG-31	White	Circular	Mucoid	Transparent	Entire	Smooth	-ve	Rod
WUSFDG-32	White	Circular	Mucoid	Transparent	Entire	Smooth	-ve	Rod
WUSFDG-33	White	Circular	Mucoid	Transparent	Entire	Smooth	-ve	Rod

Key: CC – Colony Color, CF – Colony Form, CT – Colony Texture, O – Opacity, CM – Colony Margin, A – Appearance, GS – Gram Staining, SB – Shape of Bacterium.

Twelve rhizobia isolates were selected for further characterization based on their responses to Congo Red (YEMA-CR) and Bromothymol Blue (YEMA-BTB) tests. In the YEMA-CR medium, these isolates did not absorb the Congo red dye after three days of incubation at 28°C, forming creamy-colored colonies. This behavior is typical of *Rhizobium* species, which are known to resist dye absorption, thereby helping differentiate them from other fast-growing soil bacteria such as *Agrobacterium spp*., which tend to absorb the dye and appear red. In the YEMA-BTB test, all twelve isolates changed the deep green color of the medium to yellow, indicating acid production from carbohydrate metabolism. This is a key trait of fast-growing rhizobia (e.g., *Rhizobium spp*.), in contrast to slow-growing strains such as *Bradyrhizobium*, which generally maintain an alkaline or neutral pH in this medium. Microscopically, all isolates were confirmed as gram-negative and rod-shaped, and colonies on YEMA were transparent to white, circular with entire margins, and mucoid in texture, all consistent with the morphological characteristics of rhizobia. To ensure culture purity, each isolate was sub-cultured three times by streaking on fresh YEMA plates. Overall, the results of dye-based tests, combined with colony morphology and microscopy, strongly suggest that these isolates belong to the genus rhizobia, and more specifically, to the fast-growing group. This preliminary categorization sets the stage for further biochemical and plant growth-promoting assessments.

### Biochemical characterization of isolates

All twelve rhizobia isolates tested WUSFSI-11, WUSFSI-21, WUSFSI-32, WUSFME-12, WUSFME-21, WUSFME-32, WUSFMC-12, WUSFMC-23, WUSFMC-31, WUSFMC-32, WUSFDG-12, and WUSFDG-23 exhibited positive motility (Fig 2), 80% of the tested isolates (WUSFDG-12, WUSFME-21, WUSFME-12, and WUSFMC-31) exhibited a red slant and yellow butt, indicating glucose-only fermented without gas or hydrogen sulfide (H₂S) production. These results were consistent with the positive control, suggesting a conserved glucose fermentation pathway among the majority of isolates. In contrast, 20% of the isolates (specifically WUSFMC-23) displayed a yellow slant and yellow butt, indicating the ability to ferment multiple sugars, glucose, lactose, and/or sucrose. All isolates were citrate-positive, starch-positive, urease-positive, gelatinase-negative, oxidase-positive, indole-negative, and catalase-positive, as illustrated in Table 3.

**Table 3:** Biochemical characterization of rhizobia isolates from different geographical locations.

Isolates	Biochemical characteristics										
	MT	UT	OT	SHT	TSIAT	IT	GT	CT	CUT	APT	NFT
SRCP17	+	+	+	+	RSYB	–	–	+	+	+	+
WUSFSI-11	+	+	+	+	RSYB	–	–	+	+	+	+
WUSFSI-21	+	+	+	+	RSYB	–	–	+	+	+	+
WUSFSI-32	+	+	+	+	RSYB	–	–	+	+	+	+
WUSFME-12	+	+	+	+	RSYB	–	–	+	+	+	+
WUSFME-21	+	+	+	+	RSYB	–	–	+	+	+	+
WUSFME-32	+	+	+	+	RSYB	–	–	+	+	+	+
WUSFMC-12	+	+	+	+	RSYB	–	–	+	+	+	+
WUSFMC-23	+	+	+	+	YSYB	–	–	+	+	+	+
WUSFMC-31	+	+	+	+	RSYB	–	–	+	+	+	+
WUSFMC-32	+	+	+	+	RSYB	–	–	+	+	+	+
WUSFDG-12	+	+	+	+	RSYB	–	–	+	+	+	+
WUSFDG-23	+	+	+	+	RSYB	–	–	+	+	+	+

Key: (+) positive result, (-) negative result, UT: urea test, MT: motility test, OT: oxidase test, SHT: starch hydrolysis test, IT: indole test, GT: gelatin test, CT: catalase test, CUT: citrate utilization test, APT: ammonium production test, NFT: nitrogen fixation test, TSIAT: triple sugar iron agar test; RSRB: red slant/red butt; YSYB: yellow slant/yellow butt.

**Fig 1:**
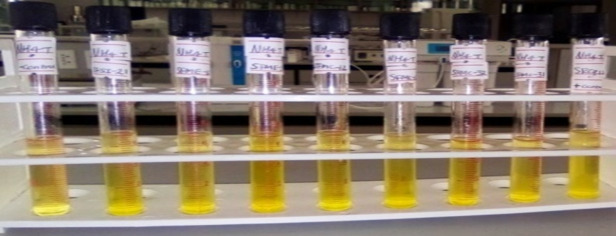
Ammonia production test of rhizobia isolates.

### Ammonia production

All tested rhizobia isolates showed positive ammonia production. Among them, 58.3% (7/12) were strong ammonia producers, exhibiting a brown-yellow color, while 41.7% (5/12) were moderate producers, indicated by a light yellow coloration (Fig 1).

### Nitrogen fixation test

**Fig 2:**
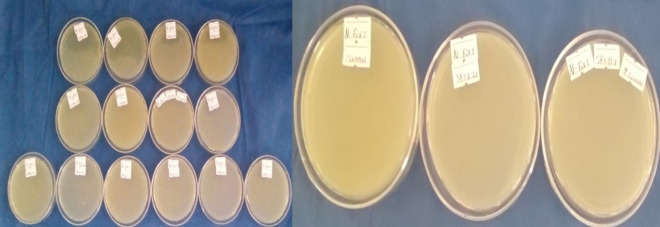
Nitrogen fixing test of rhizobia isolates.

All isolates formed a conspicuous pellicle just beneath the surface of the nitrogen-free medium after 5 days of incubation at 28 °C. This indicates that all isolates possess the ability to fix atmospheric nitrogen and grow in the absence of an external nitrogen source (Fig 2).

From the twelve rhizobia isolates, five were (WUSFDG-23, WUSFMC-31, WUSFSI-21, WUSFMC-23, and WUSFME-21) identified as potential candidates based on their efficiency in selective biochemical tests, including the oxidase test, starch hydrolysis test, and catalase test. Those isolates were further screened for their ability to promote seed germination and their symbiotic effectiveness under sterile conditions to confirm their symbiotic performance.

### Effect of rhizobia inoculation on seed germination and early seedling growth

**Fig 3:**
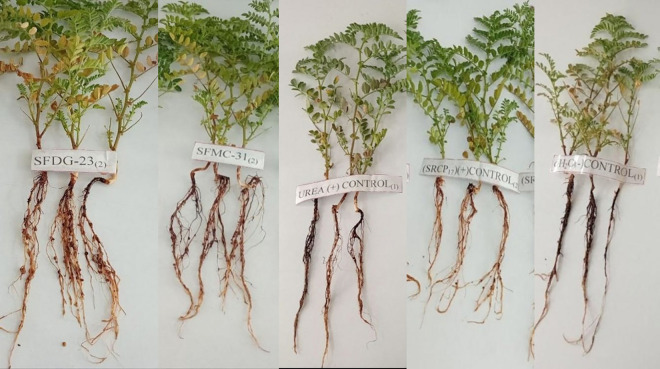
Effect of rhizobia on the growth of chickpea by pot assay.

Germination was first observed within 24 hours and continued until day 15, with rhizobia-treated seeds showing a faster and more uniform emergence pattern. After 48 hours, all seeds in both groups had germinated, but the *Rhizobium*-treated seeds exhibited a higher germination percentage compared to the non-treated control (Table 4). After two weeks of incubation at 25°C, treated seeds showed statistically significant improvements (p < 0.05) in primary root length (PRL), number of secondary roots (NSR/P), and seedling dry weight (SDW) as shown in Fig 3.

**Table 4:** Effect of rhizobia on seed germination.

Treatments	PSG	PRL (cm)	SL (cm)	SDW (g)	NSR/P	VI
SRCP17	86.67 ± 3.33^a^	25.93 ± 0.338^a^	7.20 ± 0.4509^a^	0.66 ± 0.033^a^	45.66 ± 0.8090^a^	57.46 ± 1.933^a^
WUSFDG-23	80.00 ± 5.77^ab^	25.23 ± 0.548^a^	6.76 ± 0.352^a^	0.56 ± 0.012^b^	44.4 ± 0.4359^a^	45.40 ± 3.023^b^
WUSFMC-31	73.33 ± 8.82^abc^	22.43 ± 0.617^b^	6.23 ± 0.352^ab^	0.50 ± 0.040 cd	39.93 ± 0.8762^b^	36.00 ± 1.80^bc^
WUSFSI-21	63.33 ± 8.82^c^	22.23 ± 0.913^b^	4.96 ± 0.448^bc^	0.41 ± 0.011^e^	32.13 ± 0.5487^d^	26.03 ± 3.841 cd
WUSFMC-23	70.00 ± 00^abc^	23.40 ± 0.493^b^	4.30 ± 0.472 cd	0.46 ± 0.032^de^	36.70 ± 0.2517c	27.26 ± 5.987 cd
WUSFME-21	60.00 ± 5.77^bc^	20.367 ± 0.497^c^	5.36 ± 0.393^bc^	0.53 ± 0.011^bc^	36.20 ± 0.4726^c^	31.73 ± 2.828^c^
N broth	56.67 ± 6.66^c^	15.200 ± 0.608^d^	4.00 ± 0.435^d^	0.33 ± 0.0057^f^	25.20 ± 0.5196e	18.76 ± 2.520^d^
GM	70	22.114	5.5476	0.4938	37.176	34.66
CV	15.59	4.67	13.04	7.34	2.77	17.01
LSD	19.108	1.8099	1.2669	0.0635	1.8018	10.323

Key, PSG is the percentage of seeds germinated, PRL is primary root length (cm), SL is shoot length (cm), SDW is seedling dry weight (g), NSR/P is the number of secondary roots/plants, GM is the grand mean, and VI is the vigor index.

The Pearson correlation analysis among various seedling traits revealed significant and positive relationships, indicating strong interconnections that contribute to early seedling development. Primary root length showed a very strong correlation of 89.2% with secondary root number, meaning that as the primary root grows longer, there is almost a 90% likelihood that the number of secondary roots will also increase. Similarly, it had a 76.9% positive association with seedling dry weight and a 74.3% correlation with the vigor index, suggesting that increased root length supports both greater biomass accumulation and seedling vigor. In addition, primary root length was 60.7% correlated with shoot length and 64.4% with germination percentage, indicating its broader influence on both root and shoot development.

Shoot length also exhibited strong relationships with other traits, showing an 85.3% correlation with vigor index, 78.7% with secondary root number, and 76.4% with seedling dry weight. These values demonstrate that longer shoots are consistently associated with better root development and greater biomass, reflecting the coordinated nature of above- and below-ground seedling growth. The strongest correlation observed was between secondary root number and seedling dry weight at 90.9%, indicating a very tight relationship. This suggests that increases in secondary root formation are directly and highly related to the accumulation of dry matter, which is essential for healthy seedling establishment. Germination percentage was also highly associated with vigor index, showing a correlation of 80.9%, which highlights the critical role of rapid and uniform germination in promoting strong early growth and seedling vigor. Overall, the results indicate that most of the seedling traits are highly interconnected, with correlations ranging from 60.7% to 90.9% (Table 5). These findings emphasize the importance of selecting for integrated traits such as root and shoot growth, germination rate, and biomass in breeding programs aimed at improving seedling performance and establishment under different conditions.

**Table 5:** Pearson correlation coefficient comparisons for primary root length, seedling dry weight, secondary root number, shoots length, germination percentage, and vigor index.

Correlations						
	PRL		SDW	SRN	GP	VI
PRL	1	.607^**^	.769^**^	.892^**^	.644^**^	.743^**^
SL	.607^**^	1	.764^**^	.787^**^	.720^**^	.853^**^
SDW	.769^**^	.764^**^	1	.909^**^	.537^*^	.878^**^
SRN	.892^**^	.787^**^	.909^**^	1	.674^**^	.852^**^
GP	.644^**^	.720^**^	.537^*^	.674^**^	1	.809^**^
VI	.743^**^	.853^**^	.878^**^	.852^**^	.809^**^	1

**Correlation is significant at the 0.01 level (2-tailed). *Correlation is significant at the 0.05 level (2-tailed). PRL-Primary root length, SL- SL SDW- seedling dry weight, SRN- Secondary root number, GP- Germanton percentage, VI- Vigor index.

### Symbiotic effectiveness of the rhizobia isolates in the greenhouse

After 45 days of growth, greenhouse evaluation of shoot height, nodule number, and dry matter was conducted (Table 6; Fig 3). The results revealed statistically significant differences (p < 0.05) among rhizobia isolates in terms of nodule number, nodule dry weight, and shoot dry weight. Isolate WUSFDG-23 was the most effective, producing the highest number of nodules (41.6 per plant) and the greatest nodule dry weight (0.1667 g per plant). These values were markedly higher than both control treatments: the urea-treated control, which did not form nodules due to the absence of rhizobia inoculation, and the negative control, which also showed no nodulation at all. This confirms that nodulation in chickpea plants strictly depends on inoculation with effective rhizobia isolates.

**Table 6:** Effect of Rhizobia isolates on growth parameters of chickpea on potted sterilized sand under greenhouse conditions 45 days after sowing.

Treatments	NN/p	NDW/p	SL/P	SDW/p	RL/P	RDW/P	SE (%)	SE status
UREA	0.00 ± 00^dc^	0.00 ± 00^d^	28.86 ± 0.684^c^	0.546 ± 0.015^c^	15.53 ± 0.417 cd	0.523 ± 0.018^c^	100	–
SRCP17	42.53 ± 0.46^a^	0.173 ± 0.008^a^	35.2 ± 0.55^a^	0.85 ± 0.046^a^	18.46 ± 0.64^a^	0.69 ± 0.0416^a^	156.08	HE
WUSFDG-23	41.63 ± 0.81^a^	0.166 ± 0.015^ab^	31.2 ± 0.52^b^	0.67 ± 0.032^b^	17.26 ± 0.55^ab^	0.65 ± 0.0321^ab^	121.9	HE
WUSFMC-31	41.40 ± 0.90^a^	0.16 ± 0.006^ab^	29.1 ± 0.23^c^	0.6 ± 0.03^bc^	15.9 ± 0.208^bc^	0.56 ± 0.02^bc^	109.7	HE
WUSFSI-21	36.00 ± 0.92^b^	0.133 ± 0.022^b^	26.6 ± 0.78^d^	0.42 ± 0.005^d^	15.3 ± 0.462 cd	0.51 ± 0.0306^c^	76.8	E
WUSFMC-23	33.36 ± 0.57^c^	0.123 ± 0.008^c^	26.46 ± 0.43^d^	0.57 ± 0.027^c^	14.36 ± 0.463^de^	0.5 ± 0.0231^c^	103.6	HE
WUSFME-21	34.00 ± 0.93^bc^	0.14 ± 0.017^abc^	24.5 ± 0.44^e^	0.407 ± 0.008^d^	16.46 ± 0.524^bc^	0.526 ± 0.067^bc^	74.39	E
dH2O	0.00 ± 00^dc^	0.00 ± 00^d^	21.0 ± 0.58^f^	0.23 ± 0.0057^e^	13.26 ± 0.497^e^	0.316 ± 0.068^d^	42	–
Grand mean	28.617	0.1121	27.867	0.5363	15.821	0.5346	96.7	–
CV(%)	4.12	18.75	3.41	8.30	5.31	13.41	18.3	–
LSD(*P* < 0.05)	2.039	0.034	1.6451	0.0771	1.4546	0.1241	2.12	–

Mean values in columns and rows designated with similar letters are not significantly each other, while those designated with different letters are significantly different from each other at p < 0.05. LSD (5%) = Least significant difference at p < 0.05 and CV (%) = percentage of coefficient of variation. Where SL/p = shoot length per plant, NN/p = nodule number per plant, NDW/p = nodule dry weight per plant, SDW/p = shoot dry weight per plant, LSD = least significant difference, RE = relative effectiveness, E = effective, LE = lowly effective, HE = highly effective, HE > 80%, E 50–80%, LE 35–50%, IE < 35%, the same letters are not significantly different at the P < 0.05 level, and all the data are the means of triplicates. The unit for measuring SL/p is centimeters, and the other NDW/p and SDW/p are in grams.

Among the inoculated treatments, WUSFMC-23 had the lowest nodule count (33 nodules per plant) and nodule dry weight (0.123 g per plant), though still significantly better than either control treatment. The complete absence of nodules in both controls further highlights the importance of symbiotic interaction introduced through inoculation. Shoot dry weight (SDW) varied significantly among treatments. The highest SDW (0.6667 g per plant) was recorded in plants inoculated with WUSFDG-23, which was 21.9% higher than the urea-treated control (0.5467 g) and 190% greater than the negative control (0.23 g). Even the lowest-performing inoculated isolate, WUSFME-21, with 0.4067 g SDW, still performed 74.6% better than the negative control, though it was 25.6% lower than the urea control. These results demonstrate that all *Rhizobium* inoculated treatments improved shoot dry matter accumulation compared to the controls, indicating the benefit of biological nitrogen fixation. Importantly, some isolates like WUSFDG-23 and WUSFMC-31 even surpassed the urea control, showing that they could potentially replace chemical nitrogen fertilizers.

Symbiotic effectiveness (SE), calculated based on shoot dry weight relative to the urea control, ranged from 74.3% to 121.9%. Isolates WUSFDG-23 (121.9%), WUSFMC-31 (109.7%), and WUSFMC-23 (103.6%) were classified as highly effective, meaning they stimulated shoot growth beyond that achieved by nitrogen fertilization. In contrast, WUSFSI-21 (76.8%) and WUSFME-21 (74.39%) were effective, performing better than the negative control but lower than the urea control. These results highlight that certain native rhizobia isolates, especially WUSFDG-23, possess strong potential as bio-inoculants for enhancing chickpea growth and biological nitrogen fixation in place of chemical fertilizers. Additionally, Pearson correlation analysis (Table 7) revealed significant positive associations between key traits. Shoot dry weight per plant was strongly correlated with nodule number (r = 0.533, p < 0.01), nodule dry weight (r = 0.531, p < 0.01), root length (r = 0.769, p < 0.01), root dry weight (r = 0.826, p < 0.01), and symbiotic effectiveness (r = 0.794, p < 0.01). This suggests that higher shoot biomass is consistently associated with enhanced root development and nodulation performance.

**Table 7:** Pearson correlation coefficient comparisons for nodule number, nodule dry weight, shoot dry weight, shoot length, root length per plant, root dry weight per plant, and symbiotic effectiveness.

Correlations							
	SDW	NN/P	NDW/P	SL/P	RL/P	RDW/P	SE
SDW	1	.533^**^	.531^**^	.072	.769^**^	.826^**^	.794^**^
NN/P	.533^**^	1	.976^**^	.174	.580^**^	.629^**^	.554^**^
NDW/P	.531^**^	.976^**^	1	.151	.618^**^	.648^**^	.528^**^
SL/P	.072	.174	.151	1	.055	.070	.428^*^
RL/P	.769^**^	.580^**^	.618^**^	.055	1	.885^**^	.624^**^
RDW/P	.826^**^	.629^**^	.648^**^	.070	.885^**^	1	.674^**^
SE	.794^**^	.554^**^	.528^**^	.428^*^	.624^**^	.674^**^	1

**Correlation is significant at the 0.01 level (2-tailed). *Correlation is significant at the 0.05 level (2-tailed). SDW- Shoot dry weight per plant, NN/P- Number of nodules per plant, NDW/P- Nodule dry weight per plant, SL/P- Shoot length per plant, RL/P- Root length per plant, RDW/P- Root dry weight per plant, SE- Symbiotic effectiveness.

Interestingly, shoot dry weight showed no significant correlation with shoot length (r = 0.072, p = 0.737), indicating that biomass accumulation is more dependent on root and symbiotic function than on plant height. Similarly, nodule number was highly correlated with nodule dry weight (r = 0.976), and both were positively associated with root traits and SE. Root parameters were strongly interrelated, with root length and root dry weight showing a very strong correlation (r = 0.885, p < 0.01), and both traits also contributed significantly to symbiotic effectiveness (r = 0.624 and r = 0.674, respectively). Shoot length showed a modest but significant correlation with SE (r = 0.428, p < 0.05), while its correlation with other parameters was not significant.

## Discussion

From 41 isolates, 12 isolates identified as rhizobia, following protocols of Menelih *et al*. [22], These isolates did not absorb Congo red dye and formed creamy colonies after three days at 28 °C were presumed to be authentic rhizobia, aligning with the criteria established by Hamza *et al*. [25]. The yellow color shift observed in YEMA-BTB medium indicated acid production, classifying all selected isolates as fast-growing rhizobia, in agreement with the findings of Gebremedhin [37], Nagalingam *et al.* [38], and Al-Shakarchi [39]. Microscopic analysis revealed that all isolates were gram-negative, rod-shaped bacteria, while colony morphology was transparent to white, mucoid, circular, with entire margins matching typical rhizobia characteristics as described by Nagalingam *et al*. [38]. A rigorous purification process involving three successive streaking ensured the isolation of pure cultures.

The biochemical characterization of rhizobia isolates revealed a consistent profile of enzymatic and metabolic capabilities, reflecting both conserved traits and isolate-specific adaptations. All isolates exhibited motility, as evidenced by diffuse growth in SIM medium. This finding aligns with those of Asrat *et al*. [24], confirming active motility in rhizobial populations from legume nodules. Our observation of 100% motility surpasses the 85% motility reported by Al-Shakarchi [39], possibly due to strain-specific factors or regional soil conditions influencing flagellar gene expression. The association of motility with symbiotic efficiency, as suggested by De *et al*. [40], underscores the potential of these isolates in bio-fertilizer development.

Carbohydrate fermentation profiles, assessed via the TSI test, revealed that most isolates primarily fermented glucose, consistent with *Rhizobium* strain behavior [26,27]. The uniform absence of H₂S production across isolates matches findings by Verma *et al*. [41], reaffirming that this trait is rare among agriculturally relevant rhizobia. Starch hydrolysis was variably expressed, with some isolates (WUSFME-21, WUSFMC-31, and WUSFDG-23) producing large clearing zones indicative of high amylase activity. In contrast, isolates such as WUSFSI-32, WUSFME-12, and WUSFME-32 formed smaller and less distinct clear zones, indicating comparatively lower amylase production or activity. This variability is in line with (Wanore *et al*. [42] reflects strain-specific enzymatic capabilities. Differences from the more uniform activity reported by Mir *et al*. [27] highlight the influence of ecological origin on enzyme expression. The quantitative and qualitative hydrolysis results are summarized in Table 3. All isolates showed positive citrate utilization, demonstrated by the color changes on Simmons’ citrate agar. This confirms a widespread capacity for organic acid metabolism, supporting findings by Makkar and Jangra [20]. Contrasts with Hossain *et al*. [29], who observed partial citrate utilization in acidic soils, suggest environmental modulation of metabolic gene expression.

Universal urease positivity among isolates supports the role of *Rhizobium* in nitrogen cycling, as described by Mir *et al.* [27] and Pawar *et al*. [43]. This trait’s consistency suggests adaptation to rhizosphere environments with high nitrogen turnover. All isolates tested negative for gelatinase, indicating a lack of extracellular protease production. This is consistent with Mir *et al*. [27] but contrasts with the positive gelatinase activity reported by Aung and Oo [19], possibly due to differences in protein availability or ecological pressures in marine vs. terrestrial environments. Oxidase activity varied across isolates but was universally present, suggesting aerobic metabolism supported by cytochrome c oxidase. These results corroborate Hossain *et al*. [29] and reinforce oxidase testing as a useful indicator of respiratory potential in rhizobia. Furthermore, oxidase-positive reactions suggest that the tested isolates possess active aerobic respiratory pathways, which may influence their ecological behavior, symbiotic performance, and potential suitability for bioinoculant development. The complete absence of indole production in all isolates implies a lack of tryptophanase activity. This result aligns with Shihab and Alkurtany [44] but differs from Hossain *et al*. [29], who observed weak indole production in some strains. The lack of indole biosynthesis may limit auxin production via the tryptophan-dependent pathway under laboratory conditions.

The catalase activity observed in all confirms their ability to detoxify hydrogen peroxide, a reactive oxygen species commonly encountered in soil environments and during symbiotic interactions. The vigorous bubbling, particularly in isolates WUSFDG-23, WUSFMC-32, and WUSFMC-23, indicates elevated catalase expression, suggesting enhanced oxidative stress tolerance. These findings corroborate those of Pervin *et al*. [17], who linked high catalase activity in *Rhizobium spp*. to environmental resilience and symbiotic efficiency. Conversely, the lower catalase activity reported by Pawar *et al*. [43] in saline and arid regions highlights the influence of ecological factors on gene regulation. The strong activity in our isolates may reflect adaptation to microenvironments with high organic matter turnover. Moreover, Al-Shakarchi [39] reported a positive correlation between catalase expression and nitrogen fixation efficiency, supporting the potential of catalase as a functional biomarker for selecting elite bio-inoculant strains.

Ammonia production was uniformly positive across all isolates, as evidenced by the characteristic color change in Nessler’s reagent. This result is consistent with previous studies [30,45], which link ammonia production to plant growth promotion, especially under nutrient-limited conditions. Notably, the consistently strong color development suggests robust metabolic activity across all isolates, warranting further quantitative analysis for differentiation. Additionally, all (100%) isolates formed visible pellicles in nitrogen-free medium, a hallmark of nitrogenase activity. This aligns with reports by Rodrigues *et al*. [28] and Purwaningsih *et al*. [46], emphasizing pellicle formation as an indicator of nitrogen-fixing potential. The uniform response may reflect optimal assay conditions that favor nitrogenase expression. Collectively, the catalase activity, ammonia production, and nitrogen fixation capabilities of these isolates underscore their promise as multifunctional plant growth-promoting rhizobacteria for sustainable agriculture. Overall, the biochemical profiles presented here affirm both core metabolic competencies and ecological adaptations of the tested rhizobia isolates, supporting their potential for use in targeted bio-fertilization strategies.

The present study demonstrated that rhizobia inoculation significantly enhanced seed germination and early seedling development in treated seeds compared to uninoculated controls. Germination was initiated within 24 hours, with maximum emergence observed by 72 hours. Inoculated seeds exhibited both higher germination percentages and more uniform emergence, confirming earlier reports on the beneficial role of rhizobial isolates in promoting early plant growth [43,47]. These improvements are not solely attributed to nitrogen fixation but are also linked to a suite of biological mechanisms through which *Rhizobium spp*. enhance plant growth. rhizobial isolates are known to synthesize and secrete various phytohormones, including indole-3-acetic acid (IAA), cytokinin, and gibberellins, which modulate root architecture and stimulate cell division and elongation [48,49]. The observed increase in PRL and NSR/P in inoculated seeds, particularly with strain SFDG-23, may be a direct outcome of such hormonal stimulation, which enhances water and nutrient absorption in the early growth stages.

Significant improvements (p < 0.05) in primary root length (PRL), number of secondary roots per plant (NSR/P), and seedling dry weight (SDW) were observed in inoculated treatments, particularly with strain WUSFDG-23. The resulting increase in seedling vigour index (VI) highlights *Rhizobium*’s potential in boosting early-stage biomass accumulation. This supports findings by Soboka *et al*. [50], who emphasized strain-specific variability in plant-growth promotion due to mechanisms such as nitrogen fixation and phytohormone production. Although SFDG-23 showed superior performance across most parameters. These results align with Xu *et al*. [32], who emphasized that differences in plant response may depend on specific host-microbe interactions and environmental compatibility. Such strain-dependent variations suggest the need for targeted bio-inoculant selection based on crop and site conditions.

Strong positive correlations among seedling traits further validate the importance of integrated early development. The high correlation between NSR/P and SDW (r = 0.909) echoes findings by Ali *et al*. [51], who demonstrated that enhanced root branching improves nutrient uptake and biomass accumulation. Additionally, the correlation between germination percentage and VI (r = 0.809) reinforces the idea that rapid, uniform germination is critical for competitive seedling establishment, as observed by Walia *et al.* [52].

Moreover, multivariate trait relationships emphasize the importance of holistic selection strategies in breeding. Studies like Kumar *et al*. [53] have effectively applied such methods in legume improvement programs. Deeper and faster-growing roots, as highlighted by Meza *et al*. [54], further contribute to early vigor by improving access to water and nutrients key for resilience under field conditions. The data suggest that rhizobia inoculation, particularly with high-performing strains like WUSFDG-23, holds promise not only for enhancing nodulation and nitrogen fixation in later stages but also for promoting early vigor, an essential trait for successful crop establishment.

The greenhouse pot experiment conducted at Woldia University Botanical Garden provides compelling evidence for the efficacy of native *Rhizobium* isolates in enhancing chickpea growth and symbiotic nitrogen fixation. Inoculated treatments, particularly those involving strains WUSFDG-23 and WUSFMC-31, showed significantly higher shoot dry weight, nodule number, and nodule biomass compared to uninoculated and nitrogen-fertilized controls. These results are in line with previous findings demonstrating that well-adapted native isolates can outperform commercial inoculants and synthetic nitrogen sources due to their ecological compatibility and host-specific effectiveness [35,55].

Notably, WUSFDG-23 achieved the highest shoot dry weight (0.6667 g) and nodule dry weight (0.1667 g), resulting in a symbiotic effectiveness (SE) of 121.9%, which exceeded that of the nitrogen-fertilized control. According to Somasegaran and Hoben [34], SE values exceeding 100% are indicative of highly efficient biological nitrogen fixation. This reinforces the isolate’s promise as a viable alternative to chemical nitrogen fertilizers. The complete absence of nodulation in the sterile water control confirms the dependence of nodulation and nitrogen fixation on effective rhizobia inoculation, as also noted by Tena *et al*. [56].

The Symbiotic effectiveness (SE) among the isolates ranged from 74.3% to 121.9%. Isolates WUSFDG-23 (121.9%), WUSFMC-31 (109.7%), and WUSFMC-23 (103.6%) were classified as highly effective, while WUSFSI-21 (76.8%) and WUSFME-21 (74.39%) were categorized as effective. Among isolates suggests that rhizobia strain origin and host-strain computability play crucial roles in determining symbiotic performance, as indicated in Table 6. Isolates WUSFDG-23 and WUSFMC-31, sourced from Doro Gibir and Mechare, respectively, were classified as highly effective. This supports the argument by Howieson and Dilworth [57] for the screening and utilization of native rhizobial isolates specifically adapted to native agro-ecological conditions. Their promising performance in enhancing both nodulation and biomass production makes them strong candidates for development into region-specific bio-inoculants for sustainable chickpea production in Ethiopia.

Correlation analysis highlighted strong positive relationships between shoot dry weight and root dry weight (r = 0.826), root length (r = 0.769), and symbiotic effectiveness (r = 0.794), indicating that effective symbiosis promotes balanced shoot-root development. These findings are consistent with those of Kumar *et al*. [52], who reported that improved root systems resulting from rhizobial inoculation enhance nutrient acquisition and plant growth. Furthermore, the strong correlation between nodule number and nodule dry weight (r = 0.976) suggests that nodule count may serve as a reliable phenotypic marker for assessing inoculant effectiveness, echoing observations by da Silva *et al.* [58].

Interestingly, shoot length demonstrated only a moderate correlation with symbiotic traits (r = 0.428), indicating that above-ground elongation may be influenced by other physiological or hormonal factors beyond nitrogen fixation, such as the plant’s endogenous growth regulator profile [59]. Nevertheless, the strong correlation between symbiotic effectiveness and shoot dry weight (r = 0.794) reinforces the role of SE as a key integrative metric of rhizobia performance [35].

From a sustainable agriculture perspective, these findings have important implications. First, the use of native, highly effective *Rhizobium* strains such as WUSFDG-23 and WUSFMC-31 as bio-inoculants can reduce dependence on costly and environmentally harmful chemical fertilizers. By enhancing biological nitrogen fixation, these bio-inoculants promote soil health and reduce nutrient runoff, thereby contributing to ecological sustainability [60,61]. The promotion of native inoculants tailored to local conditions supports low-input, cost-effective chickpea cultivation, a critical factor for smallholder farmers in resource-limited settings. Such innovations can directly contribute to improving yield stability, crop productivity, and food security in Ethiopia and other legume-dependent regions. The superior performance of selected native rhizobia isolates, especially WUSFDG-23 and WUSFMC-31, highlights their potential as bio-fertilizers in sustainable chickpea production. These findings support the integration of native microbial resources into soil fertility management strategies, offering an eco-friendly alternative to chemical fertilizers while addressing regional challenges in legume productivity and food security.

## Conclusion

This study successfully isolated rhizobial isolates from chickpea root nodules collected across major chickpea-growing areas around Woldia. Through a series of rigorous presumptive tests, morphological observations, cultural characteristics, and key biochemical assays were performed on the isolates. Greenhouse authentication trials further validated the symbiotic effectiveness of these isolates to be rhizobia isolates, which demonstrated significant enhancements in seed germination, nodulation, plant height, and overall biomass accumulation compared to controls. These results will show that the isolates were potentially able to promote plant growth and nodulation under controlled conditions. Collectively, the findings highlight the potential of these locally adapted rhizobia isolates as eco-friendly bio-inoculants that can contribute to sustainable chickpea production systems in Woldia and similar agroecological zones.

## Supporting information

S1 File Biochemical test. (PDF)
