# Supplementary material for: Isolation, biochemical characterization, and greenhouse authentication of chickpea (Cicer arietinum L.) rhizobia collected from some major chickpea growing areas of Woldia, North Wollo, Ethiopia
Source: PLoS One. 2025 Aug 14;20(8):e0330169. doi: 10.1371/journal.pone.0330169 (PMC12352659; doi:10.1371/journal.pone.0330169)
Supplement: S1 File — (PDF) [file pone.0330169.s001.pdf]

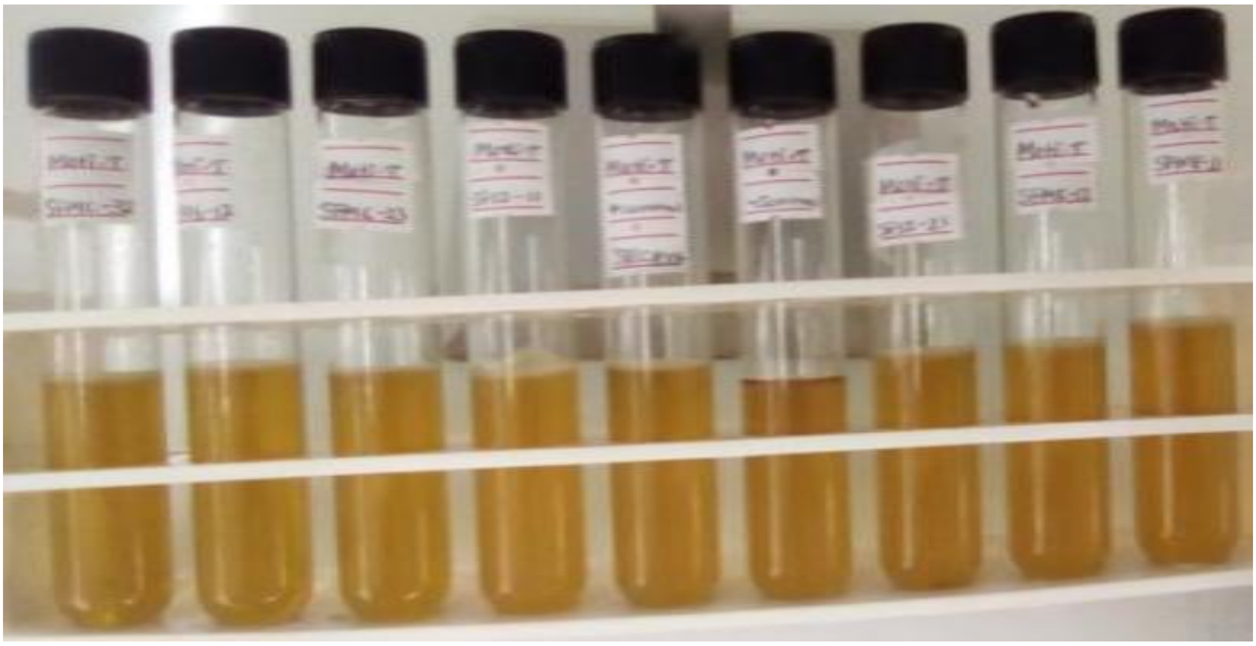

Fig: Motility test of *rhizobia* isolates

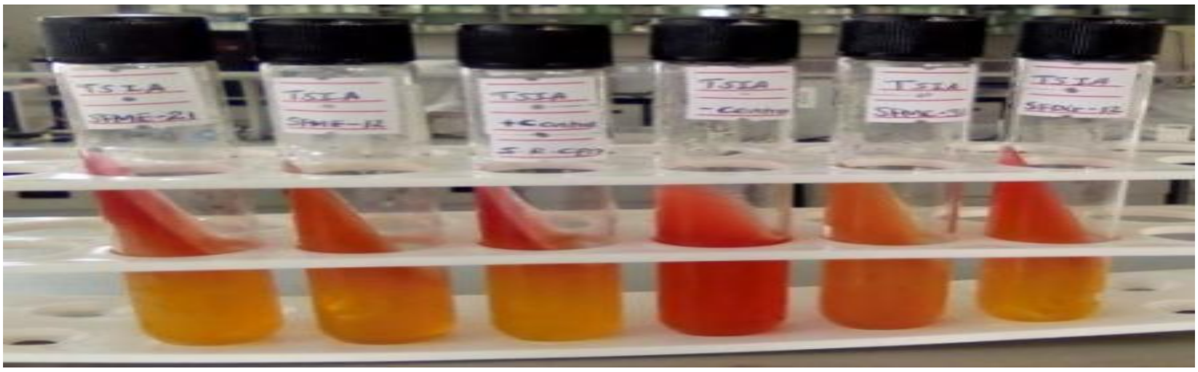

Fig: Triple sugar agar test of the *rhizobia* isolates

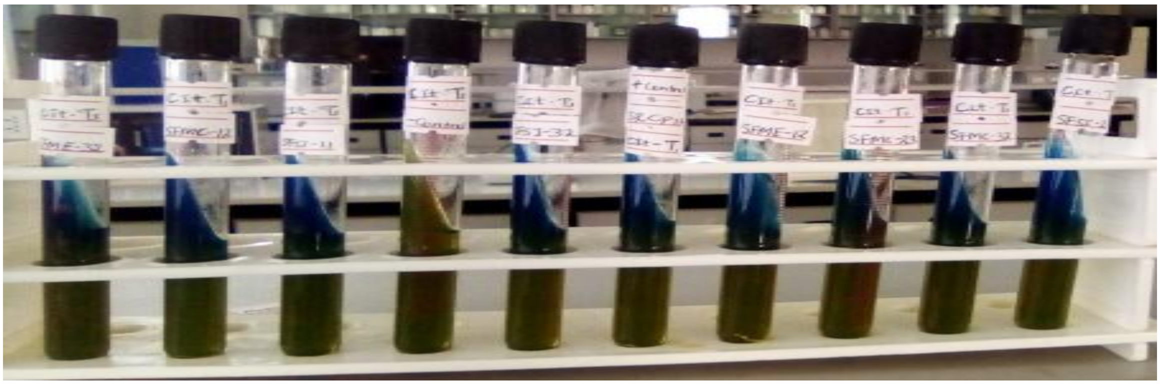

Fig: Citrate   utilization test of *rhizobia* isolates

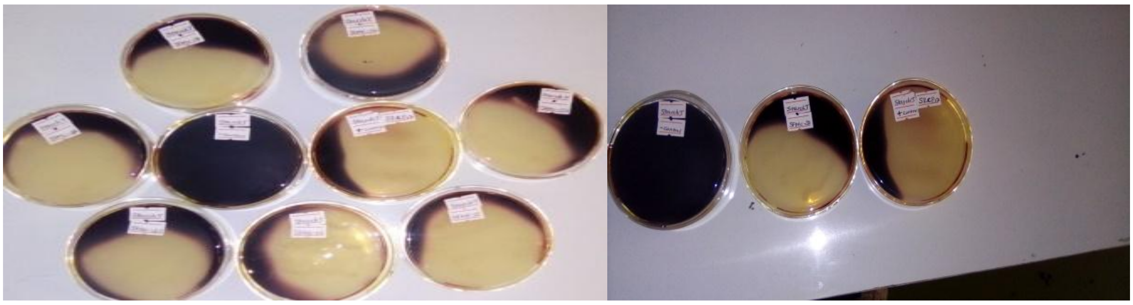

Fig: Starch hydrolysis test of *rhizobia* isolates

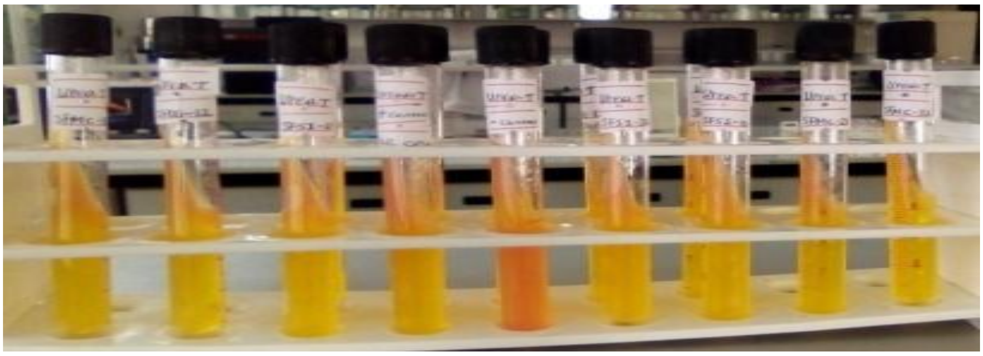

Fig: Urea test of *rhizobia* isolates

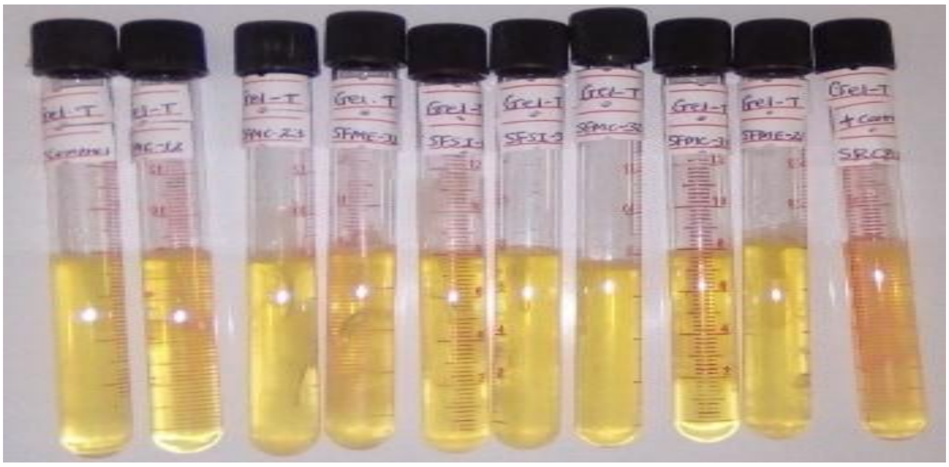

Fig: Gelatin test of *rhizobia* isolates

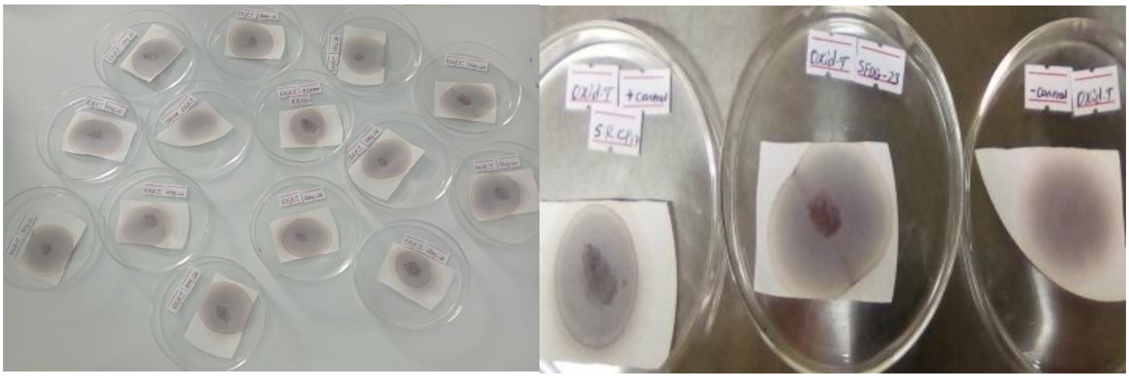

Fig1: Oxidase test of *rhizobia* isolates

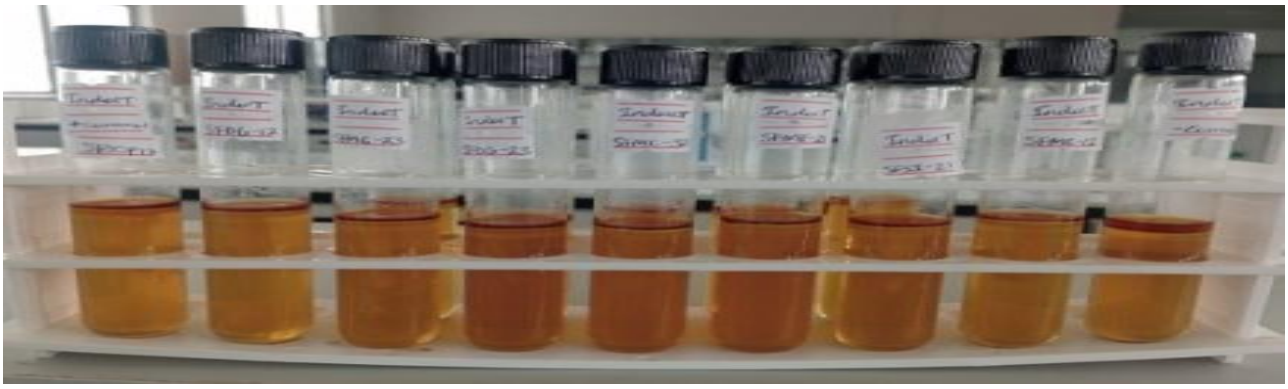

Fig: Indole test of *rhizobia* isolates

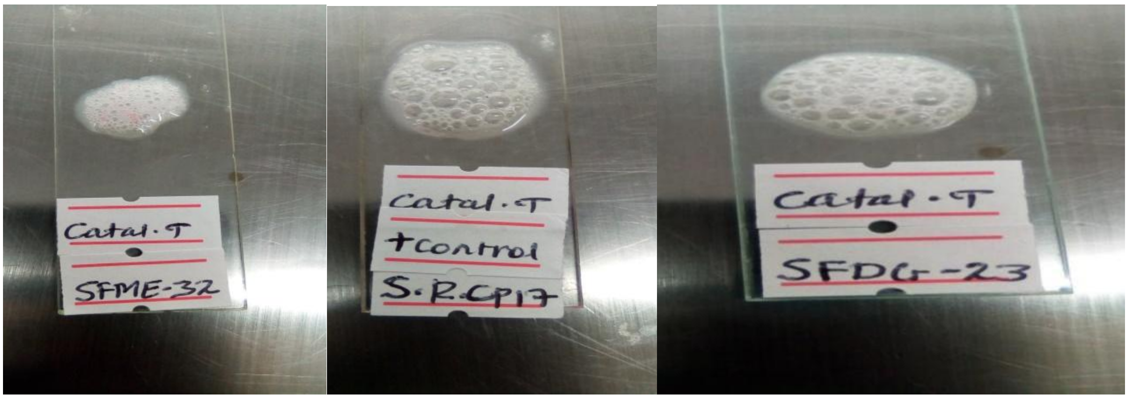

Fig: Catalyst test of *rhizobia* isolates

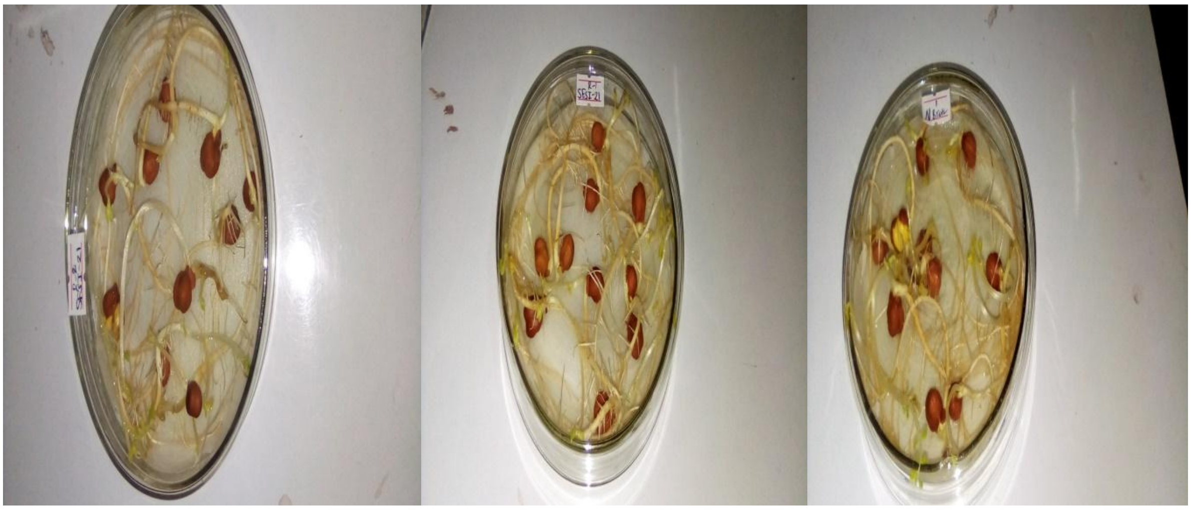

Fig : Germination tests of *Rhizobia* isolates
